# Impact of Intrapartum Antibiotic Prophylaxis on Offspring Microbiota

**DOI:** 10.3389/fped.2021.754013

**Published:** 2021-12-10

**Authors:** S. Prescott, C. Dreisbach, K. Baumgartel, R. Koerner, A. Gyamfi, M. Canellas, A. St. Fleur, W. A. Henderson, G. Trinchieri

**Affiliations:** ^1^Laboratory of Integrative Immunology, Center for Cancer Research, National Institutes of Health, Bethesda, MD, United States; ^2^College of Nursing, University of South Florida, Tampa, FL, United States; ^3^Data Science Institute, Columbia University, New York, NY, United States; ^4^School of Nursing, University of Connecticut, Storrs, CT, United States; ^5^School of Medicine, University of Connecticut, Farmington, CT, United States

**Keywords:** intrapartum antibiotics, IAP, microbiota, pregnancy, offspring dysbiosis, neonatal microbiome

## Abstract

Infants are born into a world filled with microbes and must adapt without undue immune response while exploiting the microbiota's ability to produce otherwise unavailable nutrients. The process by which humans and microbes establish this relationship has only recently begun to be studied with the aid of genomic methods. Nearly half of all pregnant women receive antibiotics during gestation to prevent maternal and neonatal infection. Though this has been largely successful in reducing early-onset sepsis, we have yet to understand the long-term consequences of antibiotic administration during gestation to developing infants. Studies involving antibiotic use in infants suggest that dysbiosis during this period is associated with increased obesity, allergy, autoimmunity, and chronic diseases in adulthood, however, research around the limited doses of intravenous antibiotics used for intrapartum prophylaxis is limited. In this mini review, we focused on the state of the science regarding the effects of intrapartum antibiotic prophylaxis on the newborn microbial colonization process. Although, the literature indicates that there is wide variety in the specific bacteria that colonize infants from birth, limited parenteral antibiotic administration prior to delivery consistently affects the microbiota of infants by decreasing bacteria in the phylum *Bacteroidetes* and increasing bacteria in the phylum *Proteobacteria*, thus altering the normal pattern of colonization that infants experience. Delivery by cesarean section and formula feeding magnify and prolong this effect. Our mini review shows that the impact of intravenous antibiotic administration during gestation has on early colonization, growth, or immune programming in the developing offspring has not been well studied in human or animal models.

## Introduction

In order to better understand the impact of intrapartum antibiotic exposure on the microbiota of offspring, one must first consider the host-microbe environment. Microbes evolved more than a billion years before the first eukaryotes, and microbes outnumber eukaryotes by an estimated 10^23^ times. Most living organisms have formed either a symbiotic or competitive relationships with them in order to survive. Often these relationships change depending on the micro and macro-environments in which each individual species inhabits. Most evidence indicates that a human infant is born nearly, if not entirely, sterile and no convincing evidence has been presented yet that bacteria are present *in utero* (1). By 4 weeks of age, the infant is colonized with 4.4 x 10^12^ bacteria, and the number will increase to a total of 3.8–4.4 x 10^13^ by adulthood (2). Bacteria are distributed on every surface interface, outnumbering human nucleated cells by roughly 10:1 (2). Interestingly, some bacterial lineages have coevolved in concert with humans, speciating as humans diverged from ancient hominids (3). Thus, bacterial niches are transferred longitudinally from mother to child exploiting the unique characteristics of the preferred habitat. Bacterially derived products for growth and development are then available to the new host and for bacterial opportunists leading to a robust environment that competes and adapts to changes as the infant progresses toward a more stable adult-like microbiota.

Infant intestinal colonization proceeds typically with early colonizers representing diverse facultative anaerobes and then increasing in strict anaerobes as the infant gut proceeds toward maturity (4). It takes nearly 3 years for the infant gut to resemble the adult colonization pattern. Though the initial bacterial inoculum occurs at delivery through maternal vaginal, fecal, skin and environmental exposure, the microbiota of identical twins is no more similar than that of fraternal twins (5). An individual's microbiota resembles the microbial ecology of those living in close proximity more than those living separately. This supports the notion that bacterial colonization is not genetically imposed, but opportunistic and proceeds differently depending on local niche conditions. Species that initially colonize the infant intestinal tract originate from the mother and the environment in equal portions, whereas the oral microbiota is shared almost entirely with the mother during the first several days of life (4). These early colonizers are species of low abundance in the maternal biome and are transient as infants settle into distinct infant colonization patterns that only gradually resolve into stable adult microbiotas. This is likely a result of environmental differences in the infant such as the increased pH of infant body cavities and exclusive human mother's milk feeding. Still, the infant microbiota remains more similar to the mother than to other adults, especially in the intestinal tract where maternal strains have an ecologic advantage and remain stable over time (4). The microbiota that inhabit infants is distinct from the adult microbiota, however, and performs specific functions that change as the infant matures (5). Thus, the infant microbiota is seeded with some stable species from the mother and expands over time with a series of microbes present locally and adapted to infant-specific microenvironments. Pioneering microbes colonize the infant in a predictable pattern and are poised to impact the developing host before settling into niche-specific adult colonization patterns within the first few years of life (6). Vaginally delivered infants born at term and breastfed are typically colonized first with facultative anaerobes mostly in the family, Enterobacteriaceae followed by obligate anaerobes such as Bifidobacterium, Bacteroides, and Clostridium (7, 8). After the introduction of solid foods, early colonizers are replaced by members of the Lachnospiracaea and Ruminococcaceae families until at ~3 years of age, when the microbiota resembles the mother and other adults in the immediate environment (6, 9, 10).

Although the maternal microbiota is strongly linked to the infant's, the duration of pregnancy, delivery mode, feeding practices, and antibiotic exposure all influence the microbial colonization of infants during this critical developmental period (11, 12). Epidemiological studies suggest an increased risk of childhood asthma, obesity, allergy, and inflammatory bowel diseases associated with delivery by cesarean section (13–16). Whether the aforementioned associations are the result of delivery mode alone or in combination with antibiotic use has not been determined as the vast majority of infants born of cesarean section receive antibiotic prophylaxis before delivery.

## Recommendations for Antibiotic Prophylaxis at Delivery

Surgical prophylaxis affects 32% of all U.S. births and reduces the incidence of post-surgical infection by 60–70% (17, 18). The American College of Obstetricians and Gynecologists recommends that all cesarean deliveries be preceded by antibiotic prophylaxis administered within an hour of delivery. Addition of a second antibiotic is considered if the cesarean is non-elective. Vaginal cleansing with povidone-iodine or low-alcohol chlorhexidine gluconate is considered before cesarean if the woman is in labor or has ruptured membranes. For patients with preterm rupture of membranes <35 weeks of gestation, a 2 day course of intravenous ampicillin and erythromycin followed by a 5 day course of oral amoxicillin and erythromycin is recommended (19).

*Streptococcus agalactiae* (Group B Streptococci, or GBS) remains the leading cause of infectious morbidity and mortality in newborns. Intrapartum antibiotic prophylaxis (IAP) administered to all GBS positive women has resulted in an 80% reduction in early onset neonatal sepsis (20). The Centers for Disease Control and Prevention (CDC) recommends that all pregnant women be tested by vaginal-rectal culture within 5 weeks of delivery, and that GBS positive women be treated for at least 4 h prior to delivery (20). Women in preterm labor with unknown GBS results should begin antibiotics for latency or prophylaxis and continue them for 48 h until negative culture results are obtained. In addition, IAP should be administered to any woman with a previous infant with invasive GBS disease, bacteriuria during any trimester, rupture of membranes greater than 18 h, or intrapartum fever greater than 38.0°C (100.4°F) (21). As a result of these guidelines, up to 50% of all pregnant women receive IAP (17, 22).

The advent of genomic analysis has led to the recognition that environmental factors influence gene expression and therefore impact human development, function, and behavior. One such environmental factor is the microbial milieu that influences infant colonization during early life. The postnatal colonization process potentially affects programming of epithelial barrier function, gut and immune homeostasis, neurobehavioral development, and angiogenesis (23). Antimicrobials administered to the mother during pregnancy may alter the maternal and infant microbiota initiating long-term effects on infant immunity, metabolism, and behavior. To date, research has largely focused health effects in children and adults resulting from microbiota perturbations as a consequence of ingested antibiotic administration during early childhood. While studying the direct effects of antimicrobial administration is important, establishing the impact of perinatal antibiotic prophylaxis, which is largely administered intravenously and of limited duration, will elucidate the effects on maternal and infant colonization, and the resultant impact on metabolic, immune, and neurobehavioral programing. This mini review will focus on research that specifically addresses the impact of intrapartum antibiotic prophylaxis on infant microbial colonization. See Table 1 for summary of selected studies.

**Table 1:** Selected studies examining perinatal antibiotics and offspring microbiota.

**Reference**	**Study design and source**	***N***	**Type of microbiome sample**	**Results**
Keski-Nisula et al. (24)	Observational, human.	45 mother-baby dyads	Maternal: vaginal Neonatal: oral	*Lactobacillus* transfer from mother to infant delayed in IAP infants.
Tormo-Badia et al. (25)	Experimental, mice.	10 mice	Fecal	Frequency of CD3+ and CD8+ T cells in the mesenteric lymph nodes were significantly higher compared with offspring.
Aloisio et al. (26)	Observational, human.	52 newborns-vaginally delivered	Fecal	*Escherichia coli, Bacteroides fragilis*, and *Bifidobacterium* were most abundant in the control group while *Lactobacilli* and *Clostridium difficile* were much lower. IAP infants had decreased *Bifidobacterium* and *Escherichia coli*.
Munyaka et al. (27)	Experimental, C57Bl/6 WT mice.	Four mice	Fecal	Fecal bacterial composition was altered at 7 weeks. Including decreased richness and modified functional pathways. Increased *Clostridium* in antibiotic group.
Aloisio et al. (28)	Observational, human.	20 term infants	Fecal	Decreased *Actinobacteria, Bacteroidetes* in the IAP group and increased *Proteobacteria* in the IAP group. Higher abundance of gram-negative phyla in IAP group.
Azad et al. (29)	Prospective longitudinal, human.	198 infants	Fecal	IAP infants deficient in *Bacteroidetes*. *Firmicutes* and *Proteobacteria* higher in IAP group. Genus *Parabacteroides* and *Bacteroides* underrepresented at 3 months in all IAP categories. Differences persistent for 1 year for emergent cesarean delivery only.
Cassidy-Bushrow et al. (30)	Observational, human.	262 infants	Fecal	If IAP, no GBS associated infant gut microbiota at 1 or 6 months. If no IAP, GBS was associated with distinct microbiota at 6 months.
Corvaglia et al. (31)	Longitudinal, human.	84 mother-infant dyads	Fecal	*Bifidobacteria* count lower in IAP group at 7 days, no difference at 30 days. Higher incidence of *Bifidobacteria* and *Lactobacillus* in both groups if exclusively human milk-fed.
Dominguez-Bello et al. (32)	Longitudinal, human.	18 mother baby dyads	Fecal	Vaginal seeding partially restores microbiota to similarity to maternal vaginal microbiota. No differences in vaginal samples based on antibiotics (2 vaginal and all C/S received IAP).
Mazzola et al. (33)	Observational, human.	26 infants	Fecal	Lower diversity in BF-IAP vs. BF-Controls. *Actinobacteria* in BF-IAP infants and absent from controls. BF-IAP dominated by *Enterobacteriacea* family mostly *Escherichia*. *Bifidobacteria* not detected in IAP infants.
Gomez-Arango et al. (34)	Observational, human.	36 mother-baby dyads	Placental, oral and fecal.	Infant microbial profiles less similar to maternal profiles if exposed to IAP. *Proteobacteria* more abundant after IAP, whole *Streptococcaceae, Gemillaceae*, and *Lactobacillus* dominated in no IAP groups.
Gonzalez-Perez et al. (35)	Experimental, C57Bl/6 mouse.	15 offspring mice.	Outcomes only	Offspring of antibiotic treated dams have fewer cytokine responses in CD8+ T cells and altered T cell receptor signaling
Miyoshi et al. (36)	Experimental, mice.	5 WT dams and 2 offspring litters per dam	Fecal	Genetically susceptible treated offspring were more susceptible to subclinical inflammation and DSS-induced colitis. Inflammatory mRNA levels are elevated in exposed mice.
Nogacka et al. (37)	Prospective longitudinal, human.	40 vaginal delivered infants	Fecal	IAP reduced levels of *Bacteroidetes* in exclusively breast-fed infants, but not formula fed infants. *Proteobacteria* and *Firmicutes* were higher in IAP exposed infants both breast and formula fed. Effect of IAP remains for at least the first months of life.
Roesch et al. (38)	Observational, human.	18 mothers	Vaginal	Higher microbial diversity in IAP groups, *Lactobacillus* greatly reduced in PCN treatment, *Pseudomonas* increased with PCN treatment.
Stearns et al. (39)	Observational, longitudinal, human.	74 infants	Fecal	Decreased *Bacteroides*, increased *Proteobacteria* in IAP groups, longer duration of ABX longer effect on microbiota.
Haupt-Jorgensen et al. (40)	Epidemiological, human.	96,840-Danish infants/children.	N/A	*In utero* antibiotics approaches significance for protecting against Type I diabetes. Early stimulation of B-cells reduces the incidence of autoimmune diabetes.
Imoto et al. (41)	Cross-sectional, human.	33 infants	Fecal	Beta diversity significantly different between antimicrobial use at delivery. Not significant by delivery mode. *Bifidobacteria* were less abundant in infants exposed to maternal antibiotics.
Nyangahu et al. (42)	Intervention, BALB/c mice.		Fecal	Decreased alpha diversity in all treated groups. Distinct community if during gestation vs. postpartum. Treated had higher proteobacteria, lower *Bacteriodetes*.

*IAP, intrapartum antibiotic prophylaxis; GBS, Group B Streptococcus; WT, wild type; DSS, dextran sulfate sodium*.

## Maternal Microbiota and Intrapartum Antibiotic Prophylaxis

Studies of the vaginal microbiota reveal that while there is no single representative vaginal microbial community, there are several common consortiums that have been labeled as “core microbiomes” (43). These consortiums tend to remain stable over time in everyone with occasional intermittent transitions to other community states depending on women's age, health, ovarian cycle, sexual activity, and reproductive state. Most of the core microbiome consortiums, or community state types (CST) are dominated by *Lactobacillus* spp. in humans, especially in Caucasian and Asian women, while Hispanic and Black women tend to have more diverse vaginal microbiotas (43). During pregnancy *Lactobacillus* species are favored, however, and CSTs dominated by other bacteria are rarely present, even in non-Caucasian women (44, 45). Although CSTs dominated by species other than *Lactobacillus* may be more common in asymptomatic non-Caucasian women, they are more likely to be associated with increased risk of bacterial vaginosis, adverse perinatal outcomes, and other infectious conditions (45, 46). It is likely that the acid-producing *Lactobacillus* species are selected during pregnancy for their pH-lowering properties that reduce colonization with more pathogenic bacterial species. The mechanism for bacterial selection in the special condition of pregnancy has not been elucidated to date but is speculatively driven by elevated estrogen leading to the accumulation of glycogen in the maturing vaginal epithelium (38).

GBS was identified in 1935, recognized as the leading cause of early onset neonatal sepsis in the 1960s, and identified as the most common cause of neonatal sepsis and meningitis in developed countries by the 1980s (47). Approximately 30% of women are colonized with GBS, and 50% will transmit the bacteria to their infants, 1% of whom will develop invasive disease. Early onset GBS-associated morbidity can be acquired by the fetus *via* vertical transmission and present as pneumonia, sepsis, or meningitis at delivery or within the first few days of life. Late onset GBS disease is usually acquired perinatally or environmentally and usually presents as meningitis. GBS disease after 90 days of age is rare (47). The introduction of IAP for GBS has reduced the incidence of early onset GBS morbidity and mortality from one in 200 births to one in 4,000 births. IAP has no effect, however, on late-onset GBS disease (48).

As IAP is administered in vaginal deliveries for the prevention of GBS disease, most studies that examined the effect of IAP on the infant microbiota examine the covariance of both antibiotic exposure and GBS colonization. Studies have shown that though IAP does reduce neonatal early-onset sepsis, it does not prevent maternal transmission of GBS, or late-onset GBS disease (38, 49, 50). In addition, GBS is found in culture-negative mothers, though in low abundance, when using culture-independent methods to examine maternal samples (38, 49). In non-pregnant women, GBS has been shown to occur in all CSTs and is associated with certain species, such as *Prevotella bivia*, within the vaginal environment (51). In pregnant women, GBS was also present in all CSTs, but in higher abundance in CSTs deficient in *Lactobacillus*. Low *Lactobacillus* CSTs were also inversely correlated with gestational age at delivery leading to the speculation that *Lactobacillus* may be protective against preterm delivery (46). As preterm infants have higher incidence and mortality from GBS EOS, preterm labor is an important indication for targeted GBS prophylaxis. Severe GBS infection was associated with loss of vaginal bacterial diversity because of the abnormal dominance of this taxa, therefore bacterial load may be of significance if using a targeted prophylactic treatment strategy (51).

IAP targets gram positive species in the case of GBS prophylaxis and both gram-positive and gram-negative species in the surgical setting. Consequently, commensal gram-positive bacteria, such as the dominant vaginal *Lactobacillus* spp., are drastically reduced after IAP (51). Gram positive gastrointestinal species such as *Bacteriodes* and *Bifidobacteia* are adversely affected as well (26, 42). With the reduction of these keystone species, opportunistic bacteria become the early colonizers and maternal vaginal bacterial communities have higher diversity after IAP (30, 38). Gomez-Arango, Barrett (34) found that infants colonized more similarly to their mothers, and with less bacterial diversity, if their mothers had not received IAP.

## Infant Microbiota

How infants become colonized is a subject of intense study. Though great variety occurs within and between individuals, metabolic pathways are stable, suggesting that local bacteria colonize the human body by exploiting and competing for the resources available in different niches (52). Colonization presents an enormous challenge to the newborn as it emerges from a protected womb to encounter bacterial, viral, and fungal antigens in their millions in addition to food antigens in milk, and immunogenic antigens in vaccines administered shortly after delivery. While amniotic fluid and colostrum favor immune tolerance toward maternal and bacterial antigens, breastmilk exerts a strong selective influence on the early microbiota (53).

The human milk matrix is highly complex and provides infants with critical immunological protection by way of non-nutritive bioactive compounds. The milk microbiome reflects thousands of years of human evolution, and neonatal exposure to these pioneering bacteria is essential for immunological maturation (54). Highly relevant to hospitalized infants, commensal bacteria stimulate antibody production (55), synthesize vitamins (56), protect against pathogens (57, 58), and stimulate intestinal angiogenesis (59).

The human milk microbiome consists largely of three dominant phyla, bifidobacterial, lactobacillus, and proteobacteria. Together, these commensal bacteria promote gut barrier function and optimizes probiotic adhesion, which inhibits pathogenic growth (60). At 1 month of age, more than 25% of the infant microbiome is derived from human milk (61). The acquisition of commensal bacteria to the breast remains an area of exploration, though recent evidence indicates vertical transmission *via* the entero-mammary pathway (62, 63). This process occurs late in pregnancy, alongside other critical biological processes that prepare the mother for birth and breastfeeding (64). Since this process occurs in the last trimester, preterm infants do not benefit from this maturation cycle, further compromising their immune potential. The absence of microbial transmission to the mammary gland leads to altered phyla or a less diverse representation in human milk (65).

Maternal exposure to antibiotics further compromises the protective potential of human milk for hospitalized infants. Intrapartum antibiotic administration is independently associated with milk microbial composition 1 month postpartum. Overall, any type of antibiotic exposure during pregnancy impedes the development of a healthy and diverse milk microbiome, namely lactobacilli and bifidobacteria (66). Infants who receive formula feedings during this critical window of immunological growth exhibit a dysbiotic intestinal environment (67). This lack of microbial diversity carries significant implications for acute infant outcomes and other serious long-term health consequences. Early breastmilk and colostrum contain not only lipids, protein, lactose, and oligosaccharides for nutrition, but large amounts of IgA, immune cells, cytokines, hormones, growth factors, and non-specific immune factors such as lysozyme and lactoferrin (60–62).

Infant fecal colonization proceeds in an orderly fashion as the gastrointestinal tract changes from an anaerobic and nearly sterile environment *in utero* to an aerobic environment with the first breathing and swallowing of air. This reverts to an anaerobic environment again at the host-bacterial interface within the first few days after the introduction of breastmilk, the passage of the first stools, and the metabolic activities of the early colonizer (68, 69). IAP may influence not only the initial seeding of the infant from the mother at delivery by altering her vaginal, skin, and anal microbiotas, but also by exerting antimicrobial properties directly through the breastmilk on the infant's oral cavity and gastrointestinal tract.

Nearly every study reviewed reported changes to the infant fecal microbiota after maternal IAP. Decreased *Bacteroidetes* and *Actinobacteria* and increased *Proteobacteria* were common after IAP in both vaginal and cesarean deliveries (28, 29, 33, 37, 42). In mice and in infants whose mothers were known to be colonized with GBS, offspring had higher *Clostridia* and *Enterococcal* colonization after IAP (26, 30); increases may be the result of the reduced *Lactobacillus* colonization seen in both animals and in humans with GBS (38, 70, 71).

As the neonatal GI tract experiences transient colonization with facultative anaerobes before assuming stable colonies of strict anaerobes, the question arises whether IAP affects early colonizers only, or whether the effects are maintained throughout these early transitions in the neonatal GI tract. Corvaglia, Tonti (31) concentrated on selected bacterial species by performing qPCR on infant fecal samples, and found that IAP resulted in lower *Bifidobacterium* counts at 7 days, but were no different at 30 days of life, with decreased counts if infants were not exclusively breast fed (31). Other studies that examined total bacterial diversity in mice and humans using 16S rDNA sequencing found altered colonization patterns lasting from 3 months to a year (29, 33, 36, 37). The combination of cesarean section and IAP or the combination of formula feeding and IAP extended the effect size and the duration of altered microbial colonization patterns (31, 33, 37, 72).

Cesarean section delivery has been linked to increased risk of allergy, asthma, obesity, autoimmune diseases, (e.g., inflammatory bowel disease) (13, 14, 73, 74). It has not been determined whether increased risks are the result of the obstetric problems that lead to cesarean delivery, the antibiotics that precede delivery, or the result of altered microbial colonization from missed vaginal exposure. Azad, Konya (29) found that all infants exposed to IAP, regardless of delivery method, had reduced fecal *Bacteroidetes* and increased *Proteobacteria* at 3 months, but that cesarean section had a larger effect size that persisted for a longer duration. Infants delivered by emergent cesarean section, and therefore exposed to obstetric problems and additional antibiotics, had dysbiosis that persisted up to a year. Others found differences in the infant microbiota based on delivery methods and demonstrated that infant colonization may be influenced by exposure to vaginal fluids administered postnatally after cesarean section. Additionally, they found no differences in maternal vaginal colonization after IAP (32). Moreover, Nyangahu, Lennard (42) also found no difference in vaginal colonization in mice 4 days after treatment with antepartum oral vancomycin. Both studies had limited sample sizes and large differences did exist in the bacterial colonization and immunoglobulins in offspring stomachs, a proxy for mouse breastmilk, supporting the notion that it is the breastmilk that is most influential in shaping the gastrointestinal microbiota of offspring after delivery (42). Stokholm, Schjørring (75) conducted a study of 738 pregnant women at 36 weeks of gestation and found changes in the vaginal microbiota after oral antibiotic exposure, but these were orally administered antibiotics prescribed for longer duration than IAP and secondary to infection (75).

The gastrointestinal microbiota has been consistently shown to exert major influences on obesity and immune system development and function (76, 77). Antibiotics administered during infancy in mice and humans increase susceptibility to allergy, asthma, and obesity, however, most studies tested orally administered antibiotics given in repeated doses similar to childhood therapy for infection or in small doses over long duration as in exposure to antibiotics in meat or water (78–81). Few studies have investigated the outcomes associated with maternal intrapartum antibiotic exposure, which is characterized by intravenous administration of short duration.

To understand the impact of perturbations to the microbiota during gestation vs. disruptions during adulthood as a result of parenterally administered antibiotics, Munyaka, Eissa (27) injected C57Bl/6 wild type mouse dams for 7 days prior to delivery with cefazolin, a 1^st^ generation cephalosporin commonly used for cesarean prophylaxis, and then exposed them to dextran sulfate sodium (DSS) at 7 weeks of age. Offspring of dams exposed to antepartum antibiotics developed earlier and more severe colitis supporting the theory that injected antenatal antibiotics can exert long-term effects on offspring GI tract immunity (27). Miyoshi, Bobe (36) treated IL-10 deficient mice neonatal and adult mice with a 4-week course of oral vancomycin, then exposed them to DSS. They found that bacterially colonized, but not germ-free offspring, exposed to antibiotics during the pre-weaning period developed persistent dysbiosis that lasted into adulthood and increased susceptibility to spontaneous and induced colitis. Early dysbiosis provoked inflammatory T cell programming and decreased regulatory T cells and anti-inflammatory mediators in the lamina propria and mesenteric lymph nodes of mice genetically predisposed to colitis (36). Germ free and adult mice exposed to antibiotics did not develop colitis, revealing that it was perturbation during early colonization that drove changes in immune programming. The same T cell programming changes occurred when non-obese diabetic mice were exposed to short courses of oral antibiotics during gestation and trended toward increased type II diabetes incidence (25). In addition, neonatal mice exposed antibiotics during gestation produced inflammatory T cells that were unable to sustain normal interferon gamma production when stimulated (35). These results indicate the microbiota derived products are required during early colonization and immune development to influence proper immune cell lineage programming and for intact signaling to elicit normal function.

The only study that focused on long-term outcomes in human infants after *in-utero* exposure to antibiotics determined after examining the records of 97,000 children, 336 of which had developed type I diabetes, that *in-utero* antibiotic use of any type or duration was not associated with childhood onset type I diabetes (40). Long-term outcomes associated with intermittent therapeutic antibiotic courses during childhood or prolonged subtherapeutic courses as in meat or water consumption have been linked to many allergic, autoimmune, and inflammatory processes as discussed earlier, but few studies have addressed long-term outcomes after brief exposure to intravenous doses as seen in IAP.

## Discussion and Conclusion

Since the advent of IAP, culture proven early onset GBS disease has declined by 68%, though decreases are not the same across all groups in the United States. Black and preterm infants continue to have a higher incidence, late onset GBS disease is unaffected, and adult onset invasive GBS disease has continued to increase substantially (82). A Cochrane Review in 2014 reported a 60–70% decrease in serious maternal post-partum infection after cesarean section when prophylactic antibiotics are administered (17). Clearly controlling the invasion of pathogenic microbes is an important part of preventative medicine for pregnant women and their infants. Until technology allows selected bacteria to be eliminated or disarmed, prophylaxis is the best strategy to reduce the risk of invasion. However, as with any medical intervention, risks and benefits must be weighed, and we currently do not know the extent of the risks involved in the prophylactic administration of antibiotics that are intravenously administered for limited duration surrounding delivery.

The most obvious first steps include: limiting cesarean sections to cases where obstetrical problems warrant it, using molecular methods to detect GBS in the delivery room, continued promotion of breastmilk, and targeting prophylaxis to heavy colonizers. Vaccine development has become a priority and in 2014 the World Health Organization began consulting on GBS vaccine development and focusing on maternal immunization (83). In addition, research must continue to determine the impact of intravenous antibiotic administration of limited duration on the maternal microbial milieu and the colonization process of offspring as most studies to date have focused on oral administration of antibiotics during infancy. Understanding the importance of exposure to vaginal and anal microbes during delivery; whether environmental, maternal skin, or breastmilk exposure drive infant colonization; if certain microbes can be restored after antibiotic exposure; and how microbes interact within the different body niches to impact the long-term growth and immunity of offspring. Answers to these questions will help determine strategies to prevent disease without negatively impacting the benefits of our long-shared history with microbes.

## Author Contributions

SP, KB, WH, and GT conceptualized the manuscript. SP, CD, MC, and AS performed the search of the literature. SP, MC, and AS created Table 1 based on the search results. SP drafted the manuscript with CD, KB, AG, MC, AS, RK, WH, and GT providing substantial revisions. SP, CD, MC, AS, and RK performed the search of the literature. All authors contributed to the article and approved the submitted version.

## Funding

This study was supported by SP: GPP Program (University of Virginia and NINR), GT: 1ZIABC011153-09, CD: F31NR017821, and WH: 1ZIANR000018.

## Conflict of Interest

The authors declare that the research was conducted in the absence of any commercial or financial relationships that could be construed as a potential conflict of interest.

## Publisher's Note

All claims expressed in this article are solely those of the authors and do not necessarily represent those of their affiliated organizations, or those of the publisher, the editors and the reviewers. Any product that may be evaluated in this article, or claim that may be made by its manufacturer, is not guaranteed or endorsed by the publisher.
